# Free-standing alumina nanobottles and nanotubes pre-integrated into nanoporous alumina membranes

**DOI:** 10.1088/1468-6996/15/4/045004

**Published:** 2014-07-18

**Authors:** Jinghua Fang, Igor Levchenko, Kostya (Ken) Ostrikov

**Affiliations:** 1 CSIRO Materials Science and Engineering, PO Box 218, Lindfield NSW 2070, Australia; 2 School of Physics, University of Melbourne, Parkville, VIC 3010, Australia; 3 School of Physics, The University of Sydney, Sydney, NSW 2006, Australia; 4 School of Chemistry, Physics, and Mechanical Engineering, Queensland University of Technology, Brisbane, QLD 4000, Australia

**Keywords:** alumina nanobottles, nanocontainers, anodization

## Abstract

A novel interfacial structure consisting of long (up to 5 *μ*m), thin (about 300 nm), highly-ordered, free-standing, highly-reproducible aluminum oxide nanobottles and long tubular nanocapsules attached to a rigid, thin (less than 1 *μ*m) nanoporous anodic alumina membrane is fabricated by simple, fast, catalyst-free, environmentally friendly voltage-pulse anodization. A growth mechanism is proposed based on the formation of straight channels in alumina membrane by anodization, followed by neck formation due to a sophisticated voltage control during the process. This process can be used for the fabrication of alumina nanocontainers with highly controllable geometrical size and volume, vitally important for various applications such as material and energy storage, targeted drug and diagnostic agent delivery, controlled drug and active agent release, gene and biomolecule reservoirs, micro-biologically protected platforms, nano-bioreactors, tissue engineering and hydrogen storage.

## 1. Introduction

Nanosized hollow structures and systems have recently attracted strong interest owing to their strong potential for targeted drug and diagnostic agent delivery [1–3], materials and energy storage [4, 5], antireflection coatings [6], gene and biomolecule reservoirs [7, 8], tissue engineering [9], microbiologically protected platforms [10], micro-bioreactors [11], for storage and controlled delivery of special chemical reagents such as, e.g., glycerol [12], corrosion inhibitors [13, 14], oxygen in aqueous media [15], guest molecules such as sugar [16], as well as other applications which require highly-functional nanosystems capable of storing bio- and organic materials and controlling their release kinetics, regulating distribution and protecting the stored material from the adverse affect of the environment. The biological and medical applications are among the most important fields which require highly controllable release of active agents to treat cancer cells without damaging ambient tissues [17]. Indeed, the drugs and active agents can be released from nanocontainers in response to changes in redox potential [18], pH [10, 19], temperature [20], and light [21]. Moreover, release of drugs and active agents is possible by fabricating nano-containers with gatekeepers sensitive to enzymes associated with a certain disease [22]. The rate of the material release depends on the shape and geometrical characteristics of nanocontainers, and also can be controlled by using nanovalves and nanogates sensitive to certain factors [19, 23]. The nanosized hollow systems are also very promising for sophisticated control of the material storage and release. In particular, the nanosized hollow hierarchical structures could be very advantageous for hydrogen storage [24] and controlled release [25] in energy-related applications.

Carbon-based nanostructures such as carbon nanotubes [26–28], fullerenes [29], nano-pipettes [30] and nanotube-based scaffolded structures (i.e. frame-like nano-morphologies with the internal hollow space) [31, 32] are among the most promising hollow nanostructures. However, carbon-based nanostructures may not be completely biocompatible with some tissues, and may also degrade under the action of certain active agents [33]. The hollow metal- and oxide-based structures such as aluminum oxide (alumina) membranes [34], metal-oxide nanobottles [35], and metal nanospheres [36] are very stable in aggressive environments and also offer new opportunities for applications.

The commonly used anodization process is a simple, cheap and fast technique, but the tunability of the anodized nanocontainers still remains a significant challenge. Importantly, an integration of the fabricated nanostructures into the device represents a major problem, since the nanocontainers are produced either in the form of free-standing (unattached) nanostructures by the templated fabrication [37], or are embedded into a solid matrix [38]. The processes based on the use of complex techniques such as plasma-enhanced chemical vapour deposition to produce high-quality structure-controllable patterns [39, 40] on top of the membrane are relatively expensive and require sophisticated equipment. At the same time, many applications require fabrication of large (up to ≈1 cm^2^) arrays of the alumina nanocontainers with highly tuneable geometrical sizes (length and container/neck diameters), partially incorporated into a supporting platform.

Ideally, these nanocontainers should be (i) attached to some bearing frame, and (ii) this frame should not interfere with the efficient use of the nanocontainers. In the ideal case, the frame should be made *of the same material as the nanocontainers*, and should be *highly porous* so not to prevent the release of the material from the nanocontainers. As an additional function, the porous frame should perform other functions, e.g., additional protection of the stored material from the microbial attack. Ultimately, all nanocontainers should be attached to the frame *during the fabrication process,* since assembly of many (up to 10^8^ cm^–2^) nanocontainers by any ‘one at a time’ technique would represent a tough technical problem.

To date, alumina nanocontainers were fabricated only in the form of internal cavities embedded in solid alumina, and stand-alone nanocontainers were produced by depositing an additional layer (e.g., carbon or oxides such as HfO_2_ and SiO_2_) onto inner surfaces of the alumina nanobottles, followed by complete alumina etching [5]. Here we report on fabrication of a *novel interfacial structure* consisting of the pattern of *stand-alone alumina nanobottles and nanotubes (nanocontainers) attached (pre-integrated) to the bearing frame of highly porous aluminum oxide* (*Al*_2_*O*_3_) *membrane*. This nanocontainers-on-frame structure is produced in an anodization/etching process and can then be easily incorporated into devices.

## 2. Experimental details

**Figure 1.**
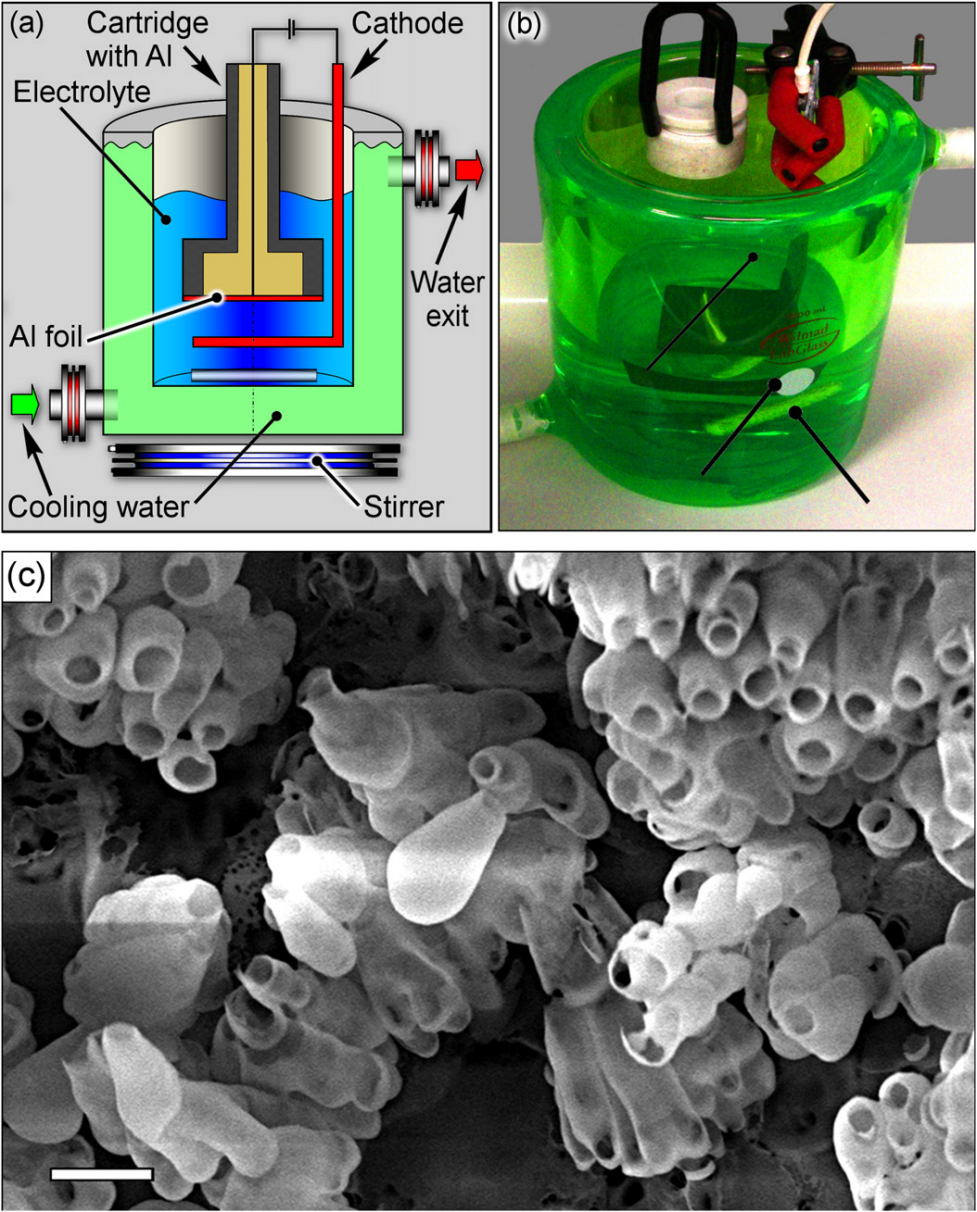
Experimental setup: (a) schematic and (b) photograph. (c) Representative SEM image of the free-standing cluster of alumina nanobottles after etching in phosphoric acid. The scale bar is 500 nm.

A schematic and photograph of the experimental setup are shown in figures 1(a) and (b), respectively, and a scanning electron microscopy (SEM) image of the typical nanobottles is shown in figure 1(c). Round samples of 1 cm in diameter made of high-purity aluminum foil (99.999%, 250 *μ*m thick) were used in all experiments. The fabrication process involves only two anodization stages. At the first stage, aluminum foil was anodized in an electrochemical anodization cell [41, 42] to form an upper nanoporous membrane and well-developed nanobottle-shaped inner cavities. Phosphoric acid (0.4 M H_3_PO_4_) was used as electrolyte for the first anodization process. During the entire anodization process, electrolyte temperature was 0 °C with an error not exceeding 0.25 °C (automatic water chiller LAUDA Alpha RA8 was used to maintain the temperature within the range with the preset error). The red and green arrows in figure 1(a) denote supply and exit of cooling water to/out of the electrochemical anodization cell. Besides, an electromagnetic stirrer SCILOGEX MS-H-Pro+ was used to stir the electrolyte during the anodization process, with a view to maintaining uniform distributions of temperature and chemical concentration in the electrolyte.

**Figure 2.**
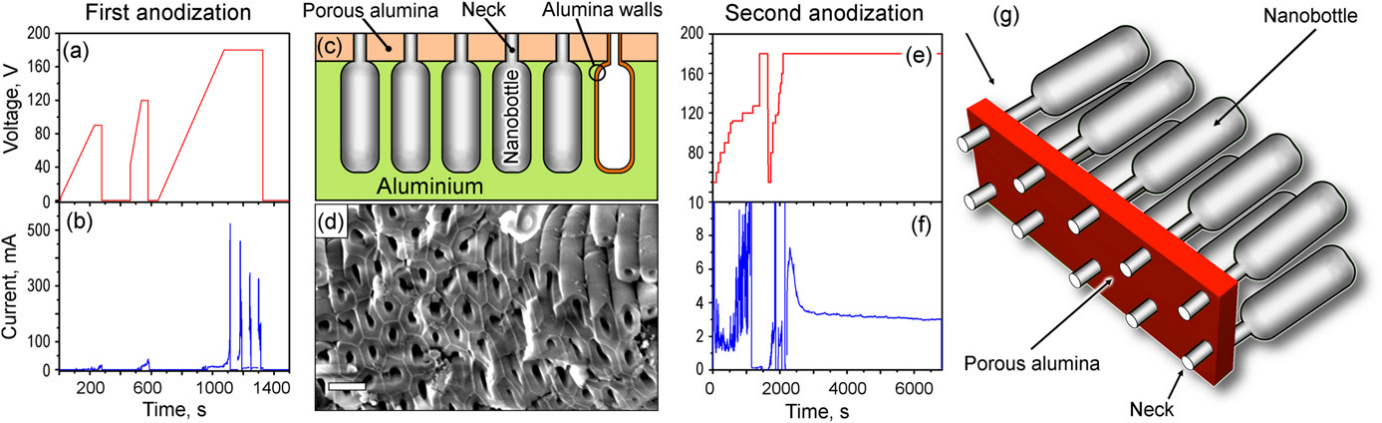
(a), (b) Time dependences of voltage and current for the first anodization step. (c) Schematic of the structure fabricated after the first anodization step, and (d) SEM image of the open nanobottle necks removal of the upper porous alumina membrane. Scale bar is 500 nm. (e), (f) Time dependences of voltage and current for the second anodization step. (g) Three-dimensional visualization of the fabricated structure.

The dependences of the voltage and current on time for the first anodization step are shown in figures 2(a) and (b). We have used a three-pulse signal, which resulted in the formation of a 1 *μ*m upper membrane of nanoporous alumina and nanobottle-shaped cavities embedded in solid alumina (figures 2(c) and (d)). Importantly, during the first anodization stage, the voltages were increased linearly with the rates of 0.375 V × s^-1^ for the first and third pulses, and 0.75 V × s^-1^ for the second pulse (figures 2(a) and (b)). The first and second voltage pulses were switched off after sudden current rises to 25 and 50 mA, respectively (a stable process current at these stages was about 10 mA). During the third pulse, the voltage rise was stopped after a very sharp current rise to 500 mA, and then the voltage was fixed at 180 V for 400 s. During this constant-voltage stage, several current spikes reaching 300–500 mA were noticed. The whole duration of the first stage was 22 min. All voltages were the optimum values for the formation of specific alumina morphologies, as described below.

Specifically, as the further examinations have shown, a nanoporous membrane was formed on the top of aluminum foil during the first (80 V) voltage pulse. Note that pores of 100–150 nm in diameter were formed due to a relatively low voltage applied and the current was maintained in the 5–10 mA range. The first current spike at 200 s was due to electrical contact between some channels and hence, increase in the alumina surface area. The sample was cooled for 3 s in the electrolyte before the second pulse application.

The second voltage pulse was used to form the branched expanding channels in the membrane (necks of the nanobottles). The pulse started from a relatively high (40 V) point to set the previous voltage level, and quickly rose to 120 V. As a result, the necks and upper parts of the nanobottles were formed due to the increased anodization voltage applied. The sample was cooled again after the second voltage pulse. The third (high-voltage) pulse was used to form the expanded channels and finally the alumina walls of long nanobottle cavities with the resultant diameter reaching 250–300 nm. Note that a lower voltage rise rate was used at this pulse to avoid current surges. Due to the above-described scheme of voltage application, nanobottle structures were formed with a typical shape shown in figure 1(c). Figure 2(d) shows a SEM image of the structure after removal of the upper nanoporous alumina membrane. From this image one can see that the nanobottle necks are hexagonally arranged and have diameters of about 100 nm.

The second anodization was the process to form free-standing nanobottles attached to the nanoporous membrane. It was started by smoothly raising a voltage to 180 V (several constant-voltage steps were made to keep the current in the limits of 10–20 mA), and then the stable 180 V was held for 1.5 h (see figures 2(e) and (f)). During this step, the phosphoric acid penetrated under the upper nanoporous membrane, and etched the solid aluminum between the walls of nanobottles formed by a thin alumina layer (note that solid aluminum is etched at these conditions much faster than alumina walls of nanobottles). As a result, all aluminum was etched away, and the stand-alone alumina nanobottles attached to the upper nanoporous membrane were formed (a three-dimensional visualization of the fabricated structure is shown in figure 2(g)). The fabricated nanobottles were characterized using a field-emission scanning electron microscope (FE-SEM, type Zeiss Auriga), equipped with an InLens secondary electron detector and operated at an electron beam energy in the range 1–5 keV. The transmission electron microscope (type JEOL 2100) was operated at an electron beam energy of 200 keV. Micro-Raman spectroscopy was conducted using a Renishaw inVia spectrometer with laser excitations of 514 and 633 nm at a spot size of ∼1 *μ*m^2^. Raman spectra from multiple spots were collected to obtain the average statistic analysis of the samples.

## 3. Results and discussion

**Figure 3.**
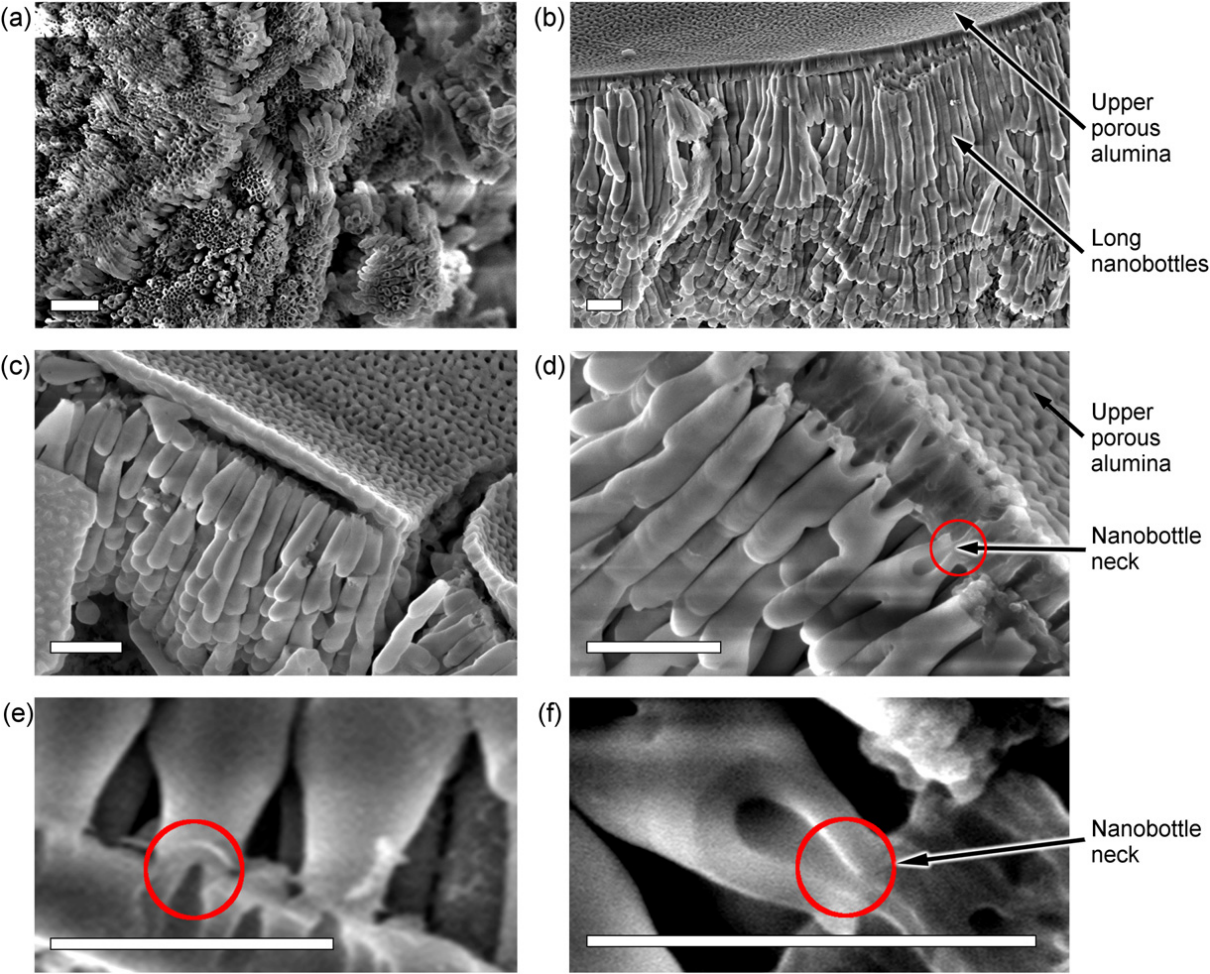
SEM images of the nanobottle + nanoporous membrane structure. Scale bars are 1 *μ*m for all panels. (a) Low-resolution SEM image illustrating a large number of free-standing alumina nanobottles after removal of the upper membrane. The nanobottles have approximately the same size and shape, they touch each other by side walls, and no other material except for the nanobottles is present. (b) SEM image of the long nanobottles attached to a thin (0.5–1.0 *μ*m) alumina membrane, side view. (c), (d) High-magnification SEM images. (d) Shows nanopores in the upper membrane and nanobottle necks connected to these nanopores. (e), (f) High-magnification SEM images showing very thin necks of the nanobottles.

Importantly, this system consists of alumina only, in contrast to other techniques which require deposition of other material (e.g., carbon) onto inner surfaces of the nanobottles [5]. Besides, all nanobottles are attached to the nanoporous membrane by their necks. Figure 3 shows results of the SEM examination of the fabricated structures.

Figure 3(a) is a low-resolution SEM image illustrating a large number of free-standing alumina nanobottles after removal of the upper membrane. It is clearly seen from this image that all nanobottles have approximately the same size and shape, they touch each other by side walls, and no other material except for the nanobottles is present. An estimated volume of a single nanobottle is of the order of 100 nanolitres (10^−7^ l).

Figure 3(b) shows SEM image of the side view of the nanobottles reaching 10–15 *μ*m in length and 500 nm in diameter, attached to a thin (0.5–1.0 *μ*m) upper nanoporous alumina membrane. Some nanobottles are branched. Figures 3(b) and (c) show the upper side of the hybrid nanobottle-membrane structure. A highly-porous surface of the upper alumina membrane can be seen in figure 3(c). Figure 3(d) is a high-resolution SEM image of the nanobottles attached to the upper membrane. From this image one can see that the pores of the upper membrane are connected to the necks of the nanobottles. Moreover, one can see that some pores of the membrane open into the inner side of the structure. Very narrow necks not exceeding 30 nm are shown in figures 3(d)–(f) and highlighted by red circles. We also point out that while the nanobottles are separated from the upper membrane on the broken sample edges (figure 3(c)), they are actually attached to the membrane in the intact samples as can be noted in figures 3(e) and (f). In these images (cut carefully not to detach the bottles), one can see the nanobottles attached to the membrane by their thin necks. More SEM images that demonstrate the nanobottle shapes and structure of the upper alumina membrane can be found in the supplementary data.

**Figure 4.**
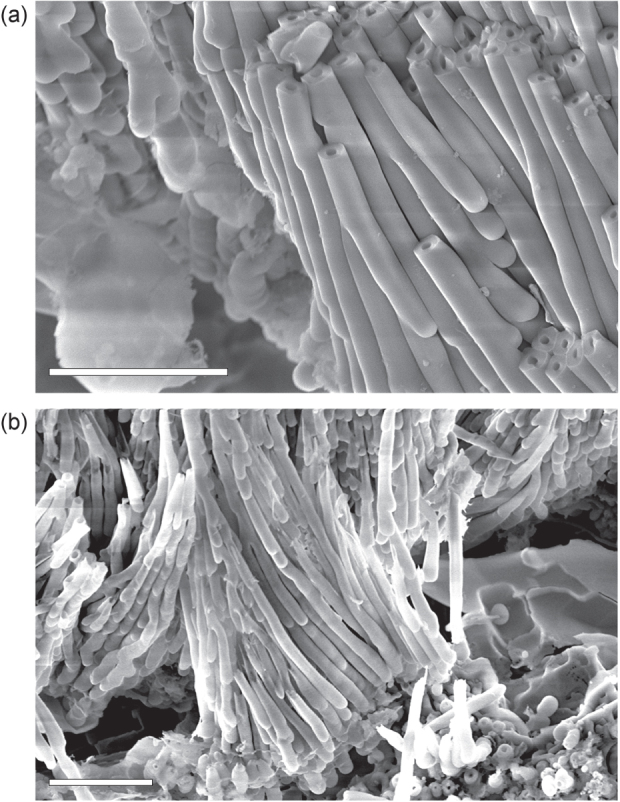
SEM images of thin nanotubes (long tubular nanocapsules). Such nanotubes demonstrate a high length-to-diameter ratio with the diameter not exceeding 200 nm. Scale bars are 2 *μ*m.

On some parts of the processed samples (specifically, at the sides) the nanobottles of slightly different shape were found. Such nanobottles (they can be called nanotubes) have higher length-to-diameter ratios with the diameter not exceeding 200 nm and the length reaching 5 *μ*m. Besides, as it can be seen in figure 4, the nanotubes have relatively thick walls reaching 1/3 of the diameter (whereas the nanobottles have thinner walls, see figure 1 and figure S1 in the supplementary data, available at stacks.iop.org/STAM/15/045004/mmedia. Moreover, the nanotubes are nearly straight and do not show many branches. We suppose that the nanotubes were formed in the sample areas with a lower current density [33].

**Figure 5.**
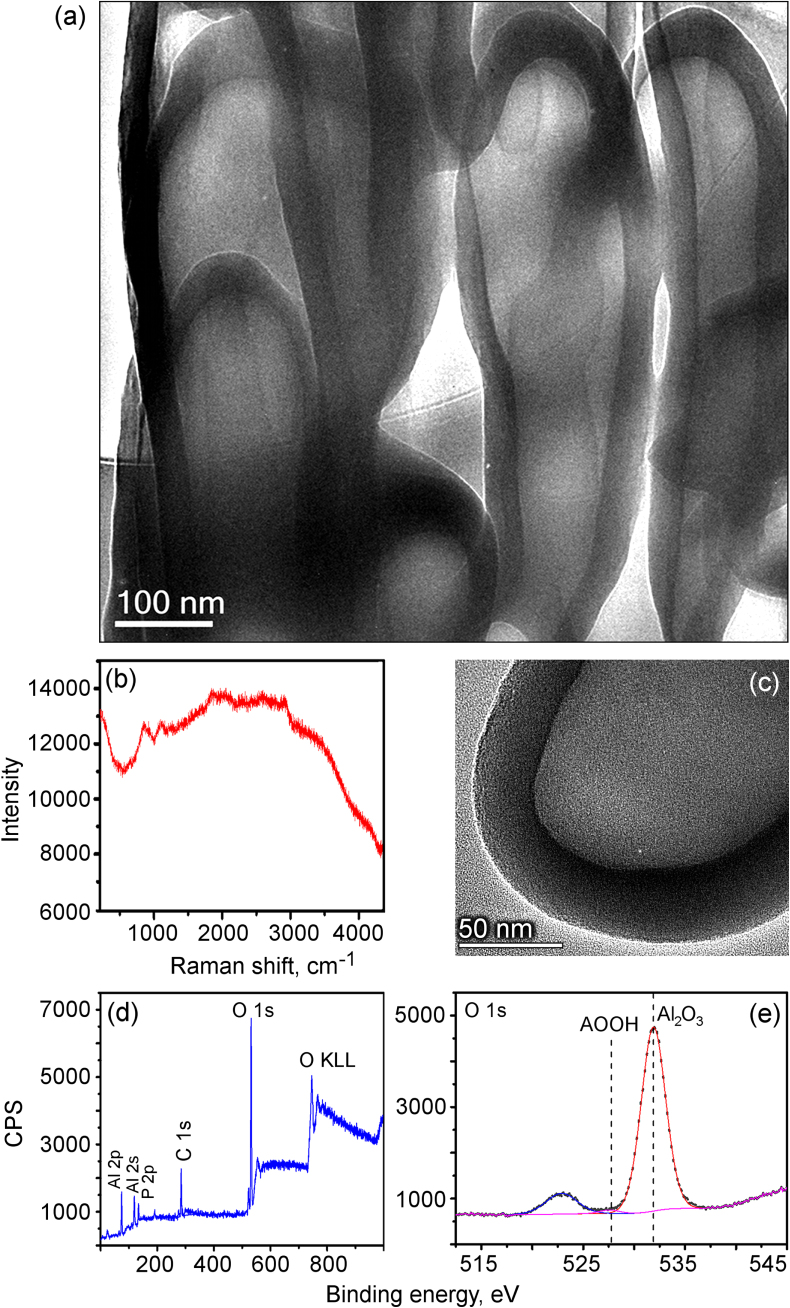
TEM, Raman and XPS characterization of the alumina nanobottles. (a) TEM images of the nanobottles; the thickness of nanobottle walls is 30–40 nm. (b) Raman characterization of the fabricated nanobottles demonstrating a typical spectrum of the amorphous alumina. (c) High-resolution TEM image shows amorphous alumina structure of the nanobottle top. (d), (e) XPS spectra showing the presence of aluminum, oxygen, and a very small amount of phosphorus.

To better characterize the fabricated structures, we have performed transmission electron microscopy (TEM), Raman, and x-ray photoelectron spectroscopy (XPS) analyses. Figure 5(a) is a TEM image of the typical nanobottles having diameters of about 150 nm. This image clearly shows that the bottles are hollow structures with the wall thickness not exceeding 30–40 nm.

Figure 5(b) is a typical Raman spectrum of the amorphous alumina structure. Figure 5(c) is a high-resolution TEM (HRTEM) image showing the amorphous alumina structure of the nanobottle top. The wall thickness is in the range of 32–40 nm. Figure S2 in the supplementary data shows TEM images of the nanobottles with different shapes and aspect ratios.

The XPS data of figures 5(d) and (e) show the presence of aluminum, oxygen, and a small amount of phosphorus in the fabricated nanobottles. More XPS results characterizing the composition of nanobottles can be found in the supplementary data file, available at stacks.iop.org/STAM/15/045004/mmedia.

## 4. Conclusions

We have demonstrated that complex nanosized hollow structures consisting of long (up to 5 *μ*m), thin (about 300 nm) alumina nanobottles attached to the rigid, thin (less than 1 *μ*m) nanoporous alumina membrane can be fabricated in a single, simple, fast, catalyst-free, environmentally friendly anodization process. These structures can be used for various applications ranging from targeted drug delivery to material storage.

## References

[C1] Gautam U K, Costa P M F J, Bando Y, Fang X, Li L, Imura M, Golberg D (2010). Recent developments in inorganically filled carbon nanotubes: successes and challenges. Sci. Technol. Adv. Mater..

[C2] Chen Y, Zheng X, Qian H, Mao Z, Ding D, Jiang X (2010). Hollow core-porous shell structure poly(acrylic acid) nanogels with a superhigh capacity of drug loading. ACS Appl. Mater. Interfaces.

[C3] Lv R, Gai S, Dai Y, Niu N, He F, Yang P (2013). Highly uniform hollow GdF_3_ spheres: controllable synthesis, tuned luminescence, and drug-release properties. ACS Appl. Mater. Interfaces.

[C4] Leyva-García S, Morallón E, Cazorla-Amorós D, Béguin F, Lozano-Castelló D (2014). New insights on electrochemical hydrogen storage in nanoporous carbons by *in situ* Raman spectroscopy. Carbon.

[C5] Pavasupree S, Suzuki Y, Pivsa-Art S, Yoshikawa S (2005). Synthesis and characterization of nanoporous, nanorods, nanowires metal oxides. Sci. Technol. Adv. Mater..

[C6] Ai P F, Liu Y L, Xiao L Y, Wang H J, Meng J X (2010). Synthesis of Y_2_O_2_S:Eu_3+_, Mg_2+_, Ti^4+^ hollow microspheres via homogeneous precipitation route. Sci. Technol. Adv. Mater..

[C7] Oh J-M, Park D-H, Choi S-J, Choy J-H (2012). LDH nanocontainers as bio-reservoirs and drug delivery carriers. Recent Pat. Nanotechnol..

[C8] Zhao X, Meng G, Han F, Li X, Chen B, Xu Q, Zhu X, Chu Z, Kong M, Huang Q (2013). Nanocontainers made of various materials with tunable shape and size. Sci. Rep..

[C9] Goldberg M, Langer R, Jia X (2007). Nanostructured materials for applications in drug delivery and tissue engineering. J. Biomater. Sci. Polym. Ed..

[C10] Kondyurin A, Levchenko I, Han Z J, Yick S, Mai-Prochnow A, Fang J, Ostrikov K, Bilek M M M (2013). Hybrid graphite film–carbon nanotube platforms for enzyme immobilization and protection. Carbon.

[C11] Chang F-P, Hung Y, Chang J-H, Lin C-H, Mou C-Y (2014). Enzyme encapsulated hollow silica nanospheres for intracellular biocatalysis. ACS Appl. Mater. Interfaces.

[C12] Suh Y J, Kil D S, Chung K S, Abdullayev E, Lvov Y M, Mongayt D (2011). Natural nanocontainer for the controlled delivery of glycerol as a moisturizing agent. J. Nanosci. Nanotechnol..

[C13] Borisova D, Möhwald H, Shchukin D G (2013). Influence of embedded nanocontainers on the efficiency of active anticorrosive coatings for aluminum alloys 2: influence of nanocontainer position. ACS Appl. Mater. Interfaces.

[C14] Tedim J, Poznyak S K, Kuznetsova A, Raps D, Hack T, Zheludkevich M L, Ferreira M G S (2010). Enhancement of active corrosion protection via combination of inhibitor-loaded nanocontainers. ACS Appl. Mater. Interfaces.

[C15] Cavallaro G, Lazzara G, Milioto S, Palmisano G, Parisi F (2014). Halloysite nanotube with fluorinated lumen: non-foaming nanocontainer for storage and controlled release of oxygen in aqueous media. J. Colloid Interface Sci..

[C16] Lee J, Lee J, Kim S, Kim C, Lee S, Min B, Shin Y, Kim C (2011). Sugar-induced release of guests from silica nanocontainer with cyclodextrin gatekeepers. Bull. Korean Chem. Soc..

[C17] Lee Z W, Zhou J, Chen C-S, Zhao Y, Tan C-H, Li L, Moore P K, Deng L-W (2011). The slow-releasing hydrogen sulfide donor, GYY4137, exhibits novel anti-cancer effects *in vitro* and *in vivo*. PLoS One.

[C18] Leung K C, Nguyen T D, Stoddart F, Zink J I (2006). Supramolecular nanovalves controlled by proton abstraction and competitive binding. Chem. Mater..

[C19] Park C, Oh K, Lee S C, Kim C (2007). Controlled release of guest molecules from mesoporous silica particles based on a pH-responsive polypseudorotaxane motif. Angew. Chem. Int. Ed..

[C20] Han Y, Shchukin D, Fernandes P, Mutihaca R-C, Möhwalda H (2010). Mechanism and kinetics of controlled drug release by temperature stimuli responsive protein nanocontainers. Soft Matter.

[C21] Nguyen T D, Leung K C-F, Liong M, Liu Y, Stoddart J F, Zink J I (2007). Versatile supramolecular nanovalves reconfigured for light activation. Adv. Funct. Mater..

[C22] Park C, Kim H, Kim S, Kim C (2009). Enzyme responsive nanocontainers with cyclodextrin gatekeepers and synergistic effects in release of guests. J. Am. Chem. Soc..

[C23] Saha S, Leung K C-F, Nguyen T D, Stoddart J F, Zink J I (2007). Nanovalves. Adv. Funct. Mater..

[C24] Wei I, Brewer J (1996). Desorption of hydrogen from palladium plating. AMP J. Technol..

[C25] Liu Z, Xue Q, Ling C, Yan Z, Zheng J (2013). Hydrogen storage and release by bending carbon nanotubes. Comput. Mater. Sci..

[C26] Keidar M, Shashurin A, Li J, Volotskova O, Kundrapu M, Zhuang T (2011). Arc plasma synthesis of carbon nanostructures: where is the frontier?. J. Phys. D: Appl. Phys..

[C27] Scheinberg D A, McDevitt M R, Dao T, Mulvey J J, Feinberg E, Alidori S (2013). Carbon nanotubes as vaccine scaffolds. Adv. Drug Deliv. Rev..

[C28] Volotskova O, Levchenko I, Shashurin A (2010). Single-step synthesis and magnetic separation of graphene and carbon nanotubes in arc discharge plasmas. Nanoscale.

[C29] Shi J, Yu X, Wang L, Liu Y, Gao J, Zhang J, Ma R, Liu R, Zhang Z (2013). PEGylated fullerene/iron oxide nanocomposites for photodynamic therapy, targeted drug delivery and MR imaging. Biomaterials.

[C30] Mani R C, Li X, Sunkara M K, Rajan K (2003). Carbon nanopipettes. Nano Lett..

[C31] Lalwani G, Kwaczala A T, Kanakia S, Patel S C, Judex S, Sitharaman B (2013). Fabrication and characterization of three-dimensional macroscopic all-carbon scaffolds. Carbon.

[C32] Levchenko I, Keidar M, Xu S, Kersten H, Ostrikov K (2013). Low-temperature plasmas in carbon nanostructure synthesis. J. Vac. Sci. Technol. B.

[C33] Kotchey G P, Zhao Y, Kagan V E, Star A (2013). Peroxidase-mediated biodegradation of carbon nanotubes *in vitro* and *in vivo*. Adv. Drug Deliv. Rev..

[C34] Fang J, Levchenko I, Ostrikov K, Prawer S (2013). Sonochemical nanoplungers: crystalline gold nanowires by cavitational extrusion through nanoporous alumina. J. Mater. Chem. C.

[C35] Cheng C-L, Chao S-H, Chen Y-F (2009). Enhancement of field emission in nanotip-decorated ZnO nanobottles. J. Cryst. Growth.

[C36] Jin Y, Gao X (2009). Spectrally tunable leakage-free gold nanocontainers. J. Am. Chem. Soc..

[C37] Gasparac R, Kohli P, Mota M O, Trofin L, Martin C R (2004). Template synthesis of nano test tubes. Nano Lett..

[C38] Lee W, Ji R, Gosele U, Nielsch K (2006). Fast fabrication of long-range ordered porous alumina membranes by hard anodization. Nat. Mater..

[C39] Ostrikov K, Neyts E C, Meyyappan M (2013). Plasma nanoscience: from nano-solids in plasmas to nano-plasmas in solids. Adv. Phys..

[C40] Mariotti D, Sankaran R M (2011). Perspectives on atmospheric-pressure plasmas for anofabrication. J. Phys. D: Appl. Phys..

[C41] Fang J-H, Spizzirri P, Cimmino A, Rubanov S, Prawer S (2009). Extremely High aspect ratio alumina transmission nanomasks: their fabrication and characterization using electron microscopy. Nanotechnology.

[C42] Fang J, Aharonovich I, Levchenko I, Ostrikov K, Spizzirri P G, Rubanov S, Prawer S (2012). Plasma-enabled growth of single-crystalline sic/alsic core-shell nanowires on porous alumina templates. Cryst. Growth Des..

